# Prevalence, Incidence, and Clearance of Anogenital Warts in Kenyan Men Reporting High-Risk Sexual Behavior, Including Men Who Have Sex With Men

**DOI:** 10.1093/ofid/ofv070

**Published:** 2015-05-12

**Authors:** Santiago Neme, Elizabeth Wahome, Grace Mwashigadi, Alexander N. Thiong'o, Joanne D. Stekler, Anna Wald, Eduard J. Sanders, Susan M. Graham

**Affiliations:** 1 Department of Medicine; 2 Departments of Epidemiology, Laboratory Medicine, and Medicine; 3 Departments of Medicine, Epidemiology, and Global Health, University of Washington, Seattle; 4 Centre for Geographic Medicine Research – Coast, Kenya Medical Research Institute, Kilifi; 5 Vaccine and Infectious Disease Division, Fred Hutchinson Cancer Research Center, Seattle, Washington; 6 Nuffield Department of Clinical Medicine, University of Oxford, Headington, United Kingdom

**Keywords:** condylomata acuminata, genital warts, HIV-1, HPV, incidence, Kenya, men, MSM

## Abstract

***Background.*** Human papillomavirus (HPV) causes a spectrum of disease, ranging from warts to cancer. Prevalence, incidence, and factors associated with anogenital warts in East African men are unknown.

***Methods.*** Kenyan men reporting high-risk sexual behavior were inspected for anogenital warts at enrollment and follow-up visits. Logistic regression was performed to identify associations with anogenital warts at baseline. Cox regression was performed to analyze predictors of incident anogenital warts, and Kaplan–Meier curves were used to estimate clearance.

***Results.*** Baseline anogenital wart prevalence in 1137 men was 2.9% (95% confidence interval [CI], 2.0%–4.0%) overall, 2.0% in human immunodeficiency virus (HIV)-uninfected men, and 9.4% in HIV-1-infected men (adjusted odds ratio, 5.43; 95% CI, 2.03–11.29). Over a median of 1.4 years, anogenital wart incidence among 1104 men was 5.3 (95% CI, 4.3–6.5) per 100 person-years. Having HIV-1 infection at baseline (adjusted hazard ratio [aHR], 1.66; 95% CI, 1.01–2.72) or a genital syndrome during follow-up (aHR, 4.78; 95% CI, 3.03–7.56) was associated with increased wart incidence. Wart clearance was lower in HIV-1-infected men (log-rank *P*<.001).

***Conclusions.*** Anogenital wart prevalence and incidence were increased in HIV-1-infected men, and anogenital warts co-occurred with other genital syndromes. Quadrivalent HPV vaccination should be recommended for young men in settings with high HIV-1 prevalence.

Genital warts are associated with significant stigma, negatively impacting the quality of life of men and women worldwide [1–3]. Treatment of persistent genital warts is challenging at best, with recurrence rates of up to 70 percent within 6 months [4]. In many resource-limited settings, wart treatment is not available, and those affected suffer without recourse. In areas of high human immunodeficiency virus (HIV)-1 prevalence, anogenital warts may be more common than elsewhere, particularly in populations with behavioral risk factors for human papillomavirus (HPV) acquisition, including multiple partners, unprotected sex, and poor genital hygiene [5–7]. Unfortunately, little is known about anogenital warts in such settings, despite the availability of 2 effective HPV vaccines (Gardasil and Gardasil-9) that protect against not only those HPV types that cause >70% of HPV-related cancers but also HPV types 6 and 11, which cause >90% of anogenital warts [8, 9].

Lavreys et al [10] estimated genital wart incidence among HIV-1-uninfected trucking company employees in Mombasa, Kenya to be 1.4 per 100 person-years. Our objective was to describe the prevalence, incidence, and factors associated with anogenital warts in a cohort of Kenyan men reporting high-risk sexual behavior, including male sex workers and men who have sex with men (MSM). We included both HIV-1-infected and uninfected men in the study, with the aim to estimate anogenital wart prevalence, incidence, and clearance in these 2 groups.

## MATERIALS AND METHODS

### Study Population

In July 2005, the Kenya Medical Research Institute (KEMRI) Human Immunodeficiency Virus/Sexually Transmitted Disease clinic opened in Mtwapa, Kenya (approximately 13 miles north of the coastal city of Mombasa), as a collaborative research site for an HIV-1 vaccine feasibility study. Since that time, HIV-1-uninfected persons reporting high-risk sexual behavior (defined as having sex with men, sex in exchange for money, recent sexually transmitted infections, serodiscordant sex partners, or multiple sex partners) have been prospectively observed and monitored for HIV-1 seroconversion. In February 2006, the clinic added a cohort study to evaluate the risk behavior and health needs of HIV-1-infected persons from the same high-risk groups. Participants were recruited through community outreach, local voluntary counseling and testing sites, and links with local lesbian, gay, bisexual, and transgender groups. Men who reported any anal sex with another man within the last 3 months were classified as MSM. All participating men, regardless of reported sexual activity, orientation, or preference, were included in the present study. Human immunodeficiency virus-1-uninfected men who acquired HIV-1 during follow-up were censored at the estimated date of infection, because data on anogenital wart detection were not available after this time point.

### Clinic Procedures

Eligible individuals were invited to receive information about participation in research and alternatives for care, including several nonresearch HIV clinics in the study area. Upon enrollment, sociodemographic and sexual behavior information was obtained with a standardized questionnaire, blood was collected for HIV-1 screening, and appropriate counseling was provided [11].

Men who reported receptive anal intercourse had monthly follow-up visits; all other participants were seen quarterly. At each follow-up visit, seronegative participants underwent HIV counseling and testing and seropositive participants underwent risk reduction counseling. Trained clinical officers obtained a medical history, performed a standardized physical examination and sexually transmitted infection (STI) screening, and provided care as indicated. Syndromic STI treatment and antiretroviral therapy, when indicated, were provided in accordance with World Health Organization and Kenyan guidelines [12]. Podophyllin was the only treatment available for warts. Participants were reimbursed KES 350 (approximately $3.70) per visit.

Trained clinical officers identified anogenital warts by visual inspection of the external genitalia. Visual inspection of the perianal area was also performed if the participant reported receptive anal sex or anorectal symptoms. Genital and perianal warts were recorded separately and included both verrucous and flat lesions. Starting in September 2006, participants who reported receptive anal sex or experienced anal symptoms were offered proctoscopy to screen for rectal lesions or discharge.

### Diagnostic Procedures

Two rapid HIV-1 test kits (Determine, Abbott Laboratories; Unigold Recombigen HIV, Trinity Biotech PLC, Ireland) were used for HIV-1 testing. Discrepant results were verified with a third-generation enzyme-linked immunosorbent assay test (Genetic System HIV-1/2 plus O EIA; Bio-Rad Laboratories, Redmond, WA). Genital syndromes diagnosed included urethritis (defined as 5 or more polymorphonuclear cells [PMNs] per high-power field on a Gram-stained urethral smear), proctitis (defined as 5 or more PMNs per high-power field on a Gram-stained rectal smear), or genital ulceration (any ulceration, either penile or perianal, on physical examination). Detection of Gram-negative, intracellular diplococci in either the urethra or rectum was used for diagnosis of gonococcal infection. All patients with laboratory-diagnosed STIs were traced and treated.

### Statistical Methods

Descriptive statistics were used to summarize baseline characteristics of the study population at cohort enrollment. The prevalence of anogenital warts in HIV-1-infected and HIV-1-uninfected men was calculated, with 95% confidence intervals (CIs), using exact binomial methods. Logistic regression was used to identify independent associations between prevalent anogenital warts (ie, the primary outcome) and potential predictors, before and after adjustment for potential confounders that were associated with anogenital warts in bivariate analysis at *P* < .10 [13]. We included HIV status and soap use as a priori predictors in multivariable analysis. Incidence rates with 95% CI were calculated using the quadratic approximation to the Poisson log likelihood for the log-rate parameter, both in the entire population and within predictor categories. Kaplan–Meier estimates were obtained, and the log-rank test was used to test for differences in risk of anogenital warts by HIV-1 status. Cox regression was then performed to analyze predictors of incident warts, with and without adjustment for potential confounders using the same model-building approach as above. Anogenital wart clearance (ie, no visible warts after previous detection) rates with 95% CI were calculated in the subgroup of men who had anogenital warts identified at enrollment or during follow-up. Kaplan–Meier estimates were obtained, and the log-rank test was used to test for differences in anogenital wart clearance by HIV-1 status. Stata/IC 11.2 was used.

### Ethics

Written, informed consent was obtained from all study participants, following procedures approved by the ethical review committees of the KEMRI (protocols 894 and 1224) and University of Washington (protocol 28292).

## RESULTS

### Population

The study was conducted between January 2005 and June 2013. Sociodemographic and other characteristics of the 1137 men in the study population are presented in Table 1. The majority of participants (59.4%) reported having sex with both men and women, 25.1% reported having sex with women only, and 15.5% reported having sex with men only. Only 26% reported formal employment, and 58% reported having been paid for sex in the past 3 months. Human immunodeficiency virus-1 prevalence at baseline was 12.1%.

**Table 1.** Baseline Characteristics of 1137 High-Risk Men in Kenya

Characteristic	n (%)
Age group	
18–24 yr	498 (44)
25–34 yr	464 (41)
>34 yr	175 (15)
Education	
Primary or lower	577 (51)
Secondary	438 (38)
Higher/tertiary	122 (11)
Ever married	
No	842 (74)
Yes	295 (26)
Employment	
None	333 (29)
Self-employed	529 (47)
Formal employment	275 (24)
Circumcised (physical examination finding)	
No	99 (9)
Yes	1035 (91)
Number of sex partners in past month^a^	
None	73 (6)
1	210 (19)
2–4	482 (42)
>4	372 (33)
Condom use for anal sex in past week^a^	
No anal sex	436 (38)
All protected	25 (2)
Any unprotected	676 (60)
Condom use for any sexual activity in past week^a^	
No sexual activity	245 (22)
All protected	231 (20)
Any unprotected	660 (58)
Group sex in past 3 mo^a^	
No	977 (86)
Yes	157 (14)
Sex work in past 3 mo^a,b^	
No	473 (42)
Yes	661 (58)
Gender of sex partners in past 3 mo^a^	
Men and women	676 (60)
Only men	176 (15)
Only women	285 (25)
Insertive anal sex in past week^a^	
No	608 (53)
Yes	529 (47)
Receptive anal sex in past week^a^	
No	587 (52)
Yes	550 (48)
Genital syndrome at current visit^a,c^	
No	659 (58)
Yes	478 (42)
Alcohol use in past month^a^	
None	81 (7)
None with sex	460 (41)
Yes with sex	593 (52)
Use of any soap in past week (self report)^a^	
No	78 (7)
Yes	1056 (93)
Soap dose in past week (self report)^a^	
None	78 (7)
1–7 times/week	352 (31)
8–14 times/week	554 (50)
15–21 times/week	136 (12)
HIV-1 status (serologic testing)	
Uninfected	999 (88)
Infected	138 (12)
Anogenital warts (physical examination finding)	
No	1104 (97)
Yes	33 (3)

Abbreviations: HIV, human immunodeficiency virus; PMNs, polymorphonuclear cells.

^a^ Updated at each visit.

^b^ Sex work was defined as having received cash, living expenses, or goods in exchange for sex.

^c^ Defined as having a diagnosis of urethritis (defined as 5 or more PMNs per high-power field on a Gram-stained urethral smear), proctitis (defined as 5 or more PMNs per high-power field on a Gram-stained rectal smear), or genital ulceration (any ulceration, either penile or perianal, on physical exam).

### Prevalence and Risk Factors for Anogenital Warts

At enrollment, 33 of the 1137 participants were diagnosed with 1 or more anogenital warts for an estimated wart prevalence of 2.9% (95% CI, 2.0%–4.0%). Twelve men had 1 or more penile warts, of whom 6 reported sex with both men and women and 6 reported sex with women exclusively. Twenty-one men had 1 or more perianal warts, of whom 17 reported sex with both women and men and 4 reported sex with men exclusively. Anogenital wart prevalence was 2.1% for men who reported sex with women exclusively, 2.3% for men who reported sex with men exclusively, and 3.4% for men who reported sex with both men and women.

In bivariate analysis, prevalent HIV-1 infection was the only variable associated with an increased prevalence of anogenital warts, with 9.4% of HIV-1-infected versus 2.0% of uninfected men having warts (odds ratio [OR], 5.09; 95% CI, 2.47–10.49; *P* < .001) (Table 2). Anogenital wart point prevalence was slightly lower among men who used soap for genital washing than those who did not (2.8% vs 3.8%), and increasing frequency of soap use showed an inverse relationship with anogenital wart prevalence (see Table 2); however, these differences were not statistically significant. In multivariate analysis including HIV-1 status and soap use as a priori predictors, only prevalent HIV-1 infection was associated with an increased anogenital wart prevalence (adjusted OR [aOR], 5.43; 95% CI, 2.03–11.29; *P* < .001).

**Table 2.** Factors Associated With Prevalent Anogenital Warts at Enrollment Among 1137 High-Risk Men in Kenya

Characteristics and Behaviors	Anogenital Wart Proportion (%)	Bivariate Analysis		Multivariate Analysis	
		Odds Ratio (95% CI)	Wald *P* Value	Adjusted Odds Ratio (95% CI)	Wald *P* Value
Age group			.48		
18–24 yr	11/498 (2.2)	Referent			
25–34 yr	16/464 (3.4)	1.58 (.73–3.44)			
>34 yr	6/175 (3.4)	1.57 (.57–4.32)			
Genital syndrome at current visit^a,b^			.14		
No	15/659 (2.3)	Referent			
Yes	18/478 (3.4)	1.68 (.84–3.36)			
HIV-1 status (serologic testing)			<.001		<.001
Uninfected	20/999 (2.0)	Referent		Referent	
Infected	13/138 (9.4)	5.09 (2.47–10.49)		5.43 (2.03–11.29)	
Circumcised (physical examination finding)			.61		
No	2/99 (2.0)	Referent			
Yes	30/1035 (2.9)	1.45 (.34–6.15)			
Education			.13		
Primary or lower	21/577 (3.6)	Referent			
Secondary	7/438 (1.6)	0.43 (.18–1.02)			
Higher/tertiary	5/122 (4.1)	1.13 (.42–3.06)			
Ever married			.56		
No	23/842 (2.7)	Referent			
Yes	10/295 (3.4)	1.25 (.59–2.66)			
Employment			.96		
None	9/333 (2.7)	Referent			
Self	16/529 (3.0)	1.12 (.49–2.57)			
Formal	8/275 (2.9)	1.08 (.41–2.83)			
Use of any soap in past week (self report)^a^			.61		.47
No	3/78 (3.8)	Referent		Referent	
Yes	30/1056 (2.8)	0.73 (.21–2.45)		0.63 (.19–2.16)	
Soap dose in past week (self report)^a^			.49		
None (did not use soap at all)	3/78 (3.8)	Referent			
1 (1–7 times/week)	12/352 (3.4)	0.88 (.24–3.20)			
2 (8–14 times/week)	17/554 (3.0)	0.79 (.23–2.77)			
3 (15–21 times/week)	1/136 (0.7)	0.19 (.02–1.81)			
Number of sex partners in past month^a^			.49		
None	2/73 (2.7)	Referent			
1	6/210 (2.9)	1.04 (.21–5.29)			
2–4	17/482 (3.5)	1.30 (.29–5.74)			
>4	8/372 (2.1)	0.78 (.16–3.75)			
Condom use for anal sex in past week^a^			.61		
No anal sex	10/436 (2.3)	Referent			
All protected	1/25 (4.0)	1.78 (.22–14.40)			
Any unprotected	22/676 (3.2)	1.43 (.67–3.06)			
Condom use for any sexual activity in past week^a^			.15		
No sexual activity	11/245 (4.5)	Referent			
All protected	8/231 (3.5)	0.76 (.30–1.93)			
Any unprotected	14/660 (2.1)	0.46 (.21–1.03)			
Group sex in past 3 months^a^			.85		
No	29/977 (2.9)	Referent			
Yes	4/157 (2.5)	0.85 (.29–2.46)			
Sex work in past 3 months^a,c^			.53		
No	12/473 (2.5)	Referent			
Yes	21/661 (3.2)	1.26 (.61–2.59)			
Gender of sex partners in past 3 months^a^			.48		
Men and women	23/676 (3.4)	Referent			
Only men	4/176 (2.3)	0.67 (.23–1.93)			
Only women	6/285 (2.1)	0.61 (.25–1.51)			
Alcohol use in past month^a^			.67		
None	1/81 (1.2)	Referent			
None with sex	14/460 (3.0)	2.51 (.32–19.36)			
Yes with sex	18/593 (3.0)	2.50 (.33–19.01)			
Insertive anal sex in past week^a^			.40		
No	20/608 (3.29)	Referent			
Yes	13/529 (2.46)	0.74 (.37–1.50)			
Receptive anal sex in past week^a^			.47		
No	15/587 (2.6)	Referent			
Yes	18/550 (3.3)	1.29 (.64–2.59)			

Abbreviations: CI, confidence interval; HIV, human immunodeficiency virus; OR, odds ratio; PMNs, polymorphonuclear cells.

^a^ Updated at each visit.

^b^ Defined as having a diagnosis of urethritis (defined as 5 or more PMNs per high-power field on a Gram-stained urethral smear), proctitis (defined as 5 or more PMNs per high-power field on a Gram-stained rectal smear), or genital ulceration (any ulceration, either penile or perianal, on physical exam).

^c^ Sex work was defined as having received cash, living expenses, or goods in exchange for sex.

### Anogenital Wart Incidence and Risk Factors

**Figure 1.**
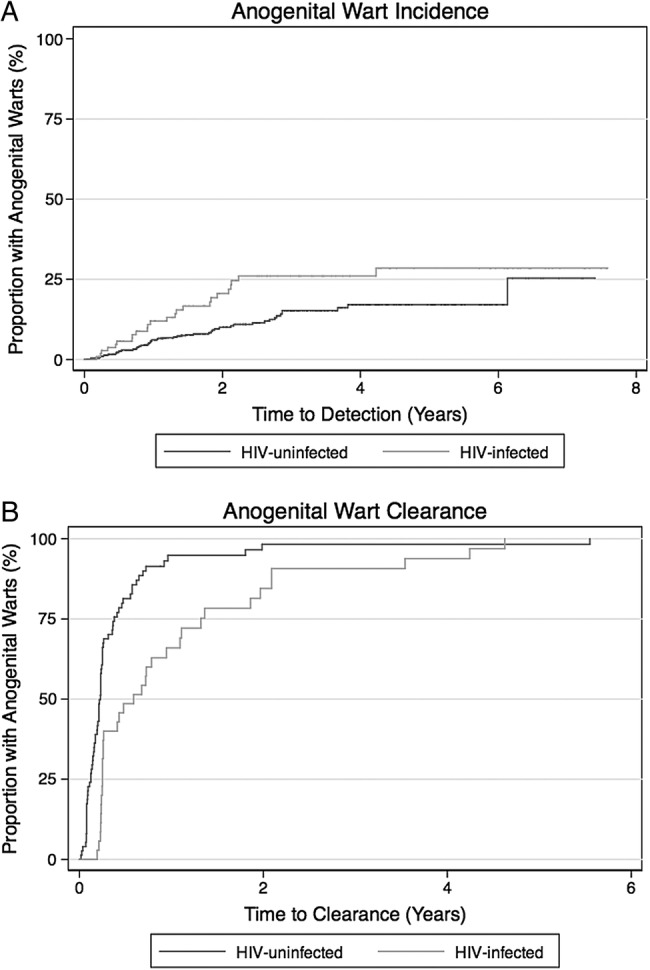
(A) Time to incident anogenital warts. Kaplan–Meier estimates for anogenital wart incidence are presented by human immunodeficiency virus (HIV)-1 status (solid line for HIV-1-infected, dashed line for HIV-1-uninfected). Anogenital wart incidence in HIV-1-infected men was 7.6 per 100 person-years (PY) versus 4.9 per 100 PY in uninfected men (log-rank *P* = .009). (B) Time to anogenital wart clearance. Kaplan–Meier estimates for anogenital wart clearance are presented by HIV-1 status (solid line for HIV-1-infected, dashed line for HIV-1-uninfected). Anogenital wart clearance in HIV-1-infected men was 96.7 per 100 PY versus 253.4 per 100 PY in HIV-1-uninfected men (unadjusted hazards ratio, 0.42; 95% confidence interval, .27–.64; *P* < .001).

The 33 men with prevalent anogenital warts on their initial exam were excluded from the incidence analysis, leaving 1104 men at risk for incident anogenital warts. Overall, follow-up time for this group was 1639 person-years (PY), with a median of 1.4 years (range, 0.03–7.59 years) and a median of 6 visits. We identified 87 men with incident anogenital warts, for an incidence rate of 5.3 per 100 PY (95% CI, 4.3–6.5 per 100 PY). Fifty men had 1 or more penile warts, of whom 34 reported sex with both men and women, 8 reported sex with men exclusively, and 8 reported sex with women exclusively. Fifty-men had 1 or more perianal warts, of whom 41 reported sex with both men and women, 12 reported sex with men exclusively, and 1 reported sex with women exclusively. A Kaplan–Meier plot of time to detection of anogenital warts (combining both penile and perianal) by HIV-1 status is shown in Figure 1A. Anogenital wart incidence in HIV-1-infected men was 7.6 per 100 PY vs 4.9 per 100 PY in uninfected men (log-rank *P* = .009).

In bivariate analysis, reporting being paid for sex in the past 3 months (hazards ratio [HR], 1.65; 95% CI, 1.06–2.57; *P* = .02) and being diagnosed with a genital syndrome at the same visit (HR, 5.10; 95% CI, 3.27–7.95; *P* < .001) were associated with an increased risk of anogenital wart acquisition (Table 3). Prevalent HIV-1 infection (HR, 1.50; 95% CI, .94–2.41; *P* = .09) and reporting sex only with male partners at the same visit (HR, 1.45; 95% CI, .79–2.64; *P* = .053) had borderline associations with increased anogenital wart acquisition, whereas being married was somewhat protective against anogenital wart acquisition (HR, 0.62; 95% CI, .37–1.04; *P* = .06). Although men who used soap for genital washing had a lower point estimate for anogenital wart incidence, this finding was not statistically significant (HR, 0.61; 95% CI, .26–1.40; *P* = .28). In the multivariate model, prevalent HIV-1 infection (aHR, 1.66; 95% CI, 1.01–2.72; *P* = .04) and being diagnosed with a genital syndrome at the same visit (aHR, 4.78; 95% CI, 3.03–7.56; *P* < .001) were both associated with an increased risk of acquiring an anogenital wart.

**Table 3.** Factors Associated With Incident Anogenital Warts Among 1104 High-Risk Men in Kenya

Characteristics and Behaviors	Warts/Per PY	Incidence/100 PY (95% CI)	Bivariate Analysis		Multivariate Analysis	
			Hazard Ratio (95% CI)	Wald *P* Value	Adjusted Hazard Ratio (95% CI)	Wald *P* Value
Age group				.10		
18–24 yr	34/485.2	7.0 (5.0–9.8)	Referent			
25–34 yr	40/803.5	5.0 (3.7–6.8)	0.71 (.45–1.12)			
>34 yr	13/358.4	3.6 (2.1–6.2)	0.52 (.27–.99)			
Genital syndrome at current visit^a,b^				<.001		<.001
No	47/1390.4	3.4 (2.5–4.5)	Referent		Referent	
Yes	40/256.7	15.6 (11.4–21.2)	5.10 (3.27–7.95)		4.78 (3.03–7.56)	
HIV-1 status (serologic testing)				.09		.04
Uninfected	63/1330.7	4.7 (3.7–6.1)	Referent		Referent	
Infected	24/316.4	7.6 (5.1–11.3)	1.50 (.94–2.41)		1.66 (1.01–2.72)	
Circumcised (physical examination finding)				.15		
No	11/130.5	8.4 (4.7–15.2)	Referent			
Yes	76/1493.3	5.1 (4.1–6.4)	0.61 (.32–1.14)			
Education				.26		
Primary or lower	39/864.0	4.5 (3.3–6.2)	Referent			
Secondary	40/624.7	6.4 (4.7–8.7)	1.45 (.93–2.26)			
Higher/tertiary	8/158.4	5.1 (2.5–10.1)	1.20 (.57–2.58)			
Ever married				.06		.23
No	68/1130.9	6.0 (4.7–7.6)	Referent		Referent	
Yes	19/516.2	3.7 (2.3–5.8)	0.62 (.37–1.04)		0.71 (.42–1.23)	
Employment				.78		
None	21/413.3	5.1 (3.3–7.8)	Referent			
Self	42/797.4	5.3 (3.9–7.1)	1.12 (.66–1.90)			
Formal	24/436.4	5.5 (3.7–8.2)	1.23 (.68–2.22)			
Use of any soap in past week (self report)^a^				.28		.49
No	6/66.2	9.1 (4.1–20.2)	Referent		Referent	
Yes	79/1567.5	5.0 (4.0–6.3)	0.61 (.26–1.40)		0.74 (.32–1.74)	
Soap dose in past week (self report)^a^				.58		
None (did not use soap at all)	6/66.2	9.1 (4.1–20.2)	Referent			
1 (1–7 times/week)	16/335.9	4.8 (2.9–7.8)	0.57 (.22–1.45)			
2 (8–14 times/week)	44/949.8	4.6 (3.4–6.2)	0.57 (.24–1.34)			
3 (15–21 times/week)	17/270.6	6.3 (3.9–10.1)	0.71 (.28–1.83)			
Number of sex partners in past month^a^				.23		
None	16/384.0	4.2 (2.6–6.8)	Referent			
1	18/473.7	3.8 (2.4–6.0)	0.85 (.43–1.68)			
2–4	29/525.5	5.5 (3.8–7.9)	1.13 (.60–2.11)			
>4	24/263.8	9.1 (6.1–13.6)	1.60 (.83–3.13)			
Condom use for anal sex in past week^a^				.21		
No anal sex	39/921.6	4.2 (3.1–5.8)	Referent			
All protected	11/100.8	10.9 (6.0–19.7)	1.90 (.96–3.78)			
Any unprotected	37/624.7	5.9 (4.3–8.2)	1.07 (.67–1.72)			
Condom use for any sexual activity in past week^a^				.47		
No sexual activity	33/640.6	5.2 (3.7–7.2)	Referent			
All protected	20/454.8	4.4 (2.8–6.8)	0.81 (.46–1.40)			
Any unprotected	34/549.1	6.2 (4.4–8.7)	1.13 (.70–1.83)			
Group sex in past 3 mo^a^				.28		
No	76/1531	5.0 (4.0–6.2)	Referent			
Yes	9/102.8	8.8 (4.6–16.8)	1.50 (.75–3.02)			
Sex work in past 3 mo^a,c^				.025		.26
No	38/1003.2	3.8 (2.8–5.2)	Referent		Referent	
Yes	47/630.7	7.5 (5.6–9.9)	1.65 (1.06–2.57)		1.31 (.82–2.11)	
Gender of sex partners in past 3 mo^a^				.053		.61
Men and women	65/1130.2	5.8 (4.5–7.3)	Referent		Referent	
Only men	13/158.8	8.2 (4.8–14.1)	1.45 (.79–2.64)		0.88 (.46–1.70)	
Only women	9/358.1	2.5 (1.3–4.8)	0.48 (.21–1.06)		0.68 (.30–1.53)	
Alcohol use in past month^a^				.36		
None	8/209.5	3.8 (1.9–7.6)	Referent			
None with sex	38/805.6	4.7 (3.4–6.5)	0.74 (.30–1.84)			
Yes with sex	39/618.7	6.3 (4.6–8.6)	1.02 (.42–2.47)			
Insertive anal sex in past week^a^				.65		
No	58/1133.3	5.1 (4.0–6.6)	Referent			
Yes	29/513.8	5.6 (3.9–8.1)	0.90 (.57–1.42)			
Receptive anal sex in past week^a^				.13		
No	49/1118.1	4.4 (3.3–5.8)	Referent			
Yes	38/529.0	7.2 (5.2–9.9)	1.40 (.90–2.16)			

Abbreviations: CI, confidence interval; HIV, human immunodeficiency virus; PMNs, polymorphonuclear cells; PY, person-years.

^a^Updated at each visit.

^b^Defined as having a diagnosis of urethritis (defined as 5 or more PMNs per high-power field on a Gram-stained urethral smear), proctitis (defined as 5 or more PMNs per high-power field on a Gram-stained rectal smear), or genital ulceration (any ulceration, either penile or perianal, on physical exam).

^c^Sex work was defined as having received cash, living expenses, or goods in exchange for sex.

### Anogenital Wart Clearance and Human Immunodeficiency Virus-1 Status

Among 120 men diagnosed with 1 or more anogenital warts, 106 had documented clearance at a subsequent visit. Median time to documented clearance was 2.7 months (interquartile range, 1.5–3.2 months). The estimated rate of clearance was 166.7 per 100 PY (95% CI, 137.7–201.8 per 100 PY). A Kaplan–Meier plot of time to anogenital wart clearance by HIV-1 status is shown in Figure 1B. Anogenital wart clearance in HIV-1-infected men was 96.7 per 100 PY vs 253.4 per 100 PY in HIV-1-uninfected men (log-rank *P* < .001).

## DISCUSSION

To our knowledge, this is the first study to report the prevalence and incidence of anogenital warts in a population of MSM and other high-risk men in Africa. We found a prevalence of 2.9% and incidence of 5.3 per 100 PY; both were substantially higher among HIV-1-infected men than HIV-1-uninfected men. Men diagnosed with a genital syndrome such as urethritis, proctitis, or genital ulceration were also at increased risk of an anogenital wart at the same visit. Soap use was not associated with decreased anogenital prevalence and incidence; however, this may have been due to the relatively small number of events, which could have limited our power to detect an association.

Our data fall within the range of global prevalence of anogenital warts (0.1%–5.1%) estimated in a 2013 systematic review by Patel et al [14]. The prevalence of anogenital warts we found in high-risk men in Kenya is similar to data from urban North American men. For example, the prevalence of genital warts in several urban US cities ranged from 2.9% to 9.2% for MSM and from 2.6% to 7.2% for men who have sex with women [15].

The anogenital wart incidence estimated in our cohort (5.3 per 100 PY) is almost 4 times higher than the anogenital wart incidence found in a cohort of male trucking company employees in Mombasa, Kenya (1.4 per 100 PY) and 23 times higher than the incidence rate of genital warts found in a multinational (United States, Mexico, Brazil) cohort of men in the general population (0.23 per 100 PY) [10, 16]. Of note, both studies excluded HIV-1-infected men. The very high overall incidence in our study even among HIV-1-uninfected men could also be due to a relatively young study population, close follow-up intervals, and frequently reported high-risk sexual behavior, including unprotected receptive anal intercourse.

In our cohort, HIV-1-infected men had higher anogenital wart prevalence and incidence compared with uninfected men, with minimal change in the magnitude of risk after adjusting for potential confounders. These results are consistent with the findings of several studies describing higher rates of HPV infection in HIV-1-infected individuals in populations across the globe [16–20], including a systematic review that found a relative risk (RR) similar to ours (1.62 vs 1.66) [21]. In a South African study, HIV-1-infected men had an increased risk of new HPV detection (any type) compared with uninfected men (RR, 2.00; 95% CI, 1.49–2.69) [22]. In addition, HIV-1-infected men had a reduced rate of HPV viral clearance relative to HIV-1-uninfected men (RR, 0.71; 95% CI, .55–.93) [22]. Human papillomavirus infection tends to persist and reactivate more frequently in HIV-infected individuals than in uninfected individuals [22]. In the present study, HIV-1 infection was associated with a decreased rate of anogenital wart clearance. Although our study is not the first to report increased anogenital wart prevalence in HIV-1-infected men, we were unable to identify any previous studies reporting anogenital wart incidence or clearance in HIV-1-infected men for comparison.

In agreement with previous studies [23], we found a significant association between incident anogenital warts and having another genital syndrome, including urethritis, proctitis, and genital ulcer disease, even after adjustment for potential confounders. Although we did not detect a significant association between prevalent anogenital warts and being diagnosed with a genital syndrome, this finding could be due to the small number of anogenital wart cases detected at enrollment or to the relatively low sensitivity of the diagnostics used for STI testing in the present study.

Because poor hygiene has been associated with increased HPV prevalence, we analyzed associations between the use of soap for genital washing and both anogenital wart prevalence and incidence [7, 24, 25]. Although we found that point estimates of the OR for prevalent anogenital warts and the HR for incident anogenital warts were less than 1, no statistically significant association was found. This is in contrast to our finding, in the same Kenyan cohort of men reporting high-risk sexual behavior, that the use of soap for genital washing was associated with a decreased risk of herpes simplex virus type 2 acquisition (adjusted incidence rate ratio, 0.3; 95% CI, .1–0.8) [26]. Additional study of the impact of hygiene on HPV viral persistence and clearance could help determine whether hygiene practices could potentially reduce the incidence of anogenital warts in this study population.

Anogenital warts are not a mere nuisance, but they can cause considerable psychological distress and morbidity [1–3]. Warts disrupt the epithelial surface and can bleed, occasionally becoming superinfected. Moreover, anogenital warts have been associated with infection by multiple HPV types (including high-risk types) and, as a result, may be associated with a long-term increased risk of anogenital cancer [27, 28]. Although it is unclear whether anogenital warts increase the risk for HIV-1 acquisition per se, 1 Kenyan study reported an increased risk of risk of HIV-1 acquisition among Kenyan men infected with HPV [29]. Other studies are currently examining the impact of genital warts on HIV incidence [30]. The quadrivalent HPV vaccine (HPV6/11/16/18) has demonstrated efficacy for the prevention of anogenital warts and anal cancer in men [9]. As evidence of the burden of HPV-related disease in African men accrues, the case for vaccination of both men and women in this region will become more and more compelling.

Strengths of this study include the longitudinal study design, the long duration of follow-up, close monitoring with monthly or quarterly visits, and a standardized physical exam including inspection for both genital and perianal warts. Our study had several limitations. First, the relatively low number of events limited our power to detect potentially important associations. In order to increase power, we created a combined variable, anogenital warts, including both genital and perianal warts. In doing this, our ability to focus on a single anatomic site was reduced. Second, we used visual inspection as our method for anogenital wart detection. Without histologic testing, it is possible that other conditions (eg, penile intraepithelial neoplasia) were misclassified as warts. However, visual inspection alone is the most frequently used method for genital wart diagnosis in several parts of the world. Finally, we were unable to test for HPV DNA and subtypes, including HPV 6/11, an important predictor of the development of warts.

## CONCLUSIONS

This population of men reporting high-risk sexual behaviors had a moderate prevalence of anogenital warts and one of the highest rates of anogenital wart acquisition ever described. We have confirmed the association between anogenital wart prevalence and HIV-1 infection and estimated a 1.7-fold increase in the risk of anogenital wart acquisition among HIV-1-infected men. We have also confirmed an association between incident anogenital wart and genital syndromes other than warts. Genital wart disease negatively impacts the quality of life of men worldwide. Treatment of persistent genital warts can be challenging, and treatment is unavailable in many resource-limited settings. The quadrivalent HPV vaccination, which has demonstrated efficacy for the prevention of anogenital warts and anal cancer in men, should be considered for young men in East Africa and in other settings with high HIV-1 prevalence.
